# Examining the effect of linkage disequilibrium on multipoint linkage analysis

**DOI:** 10.1186/1471-2156-6-S1-S83

**Published:** 2005-12-30

**Authors:** Qiqing Huang, Sanjay Shete, Michael Swartz, Christopher I Amos

**Affiliations:** 1 Department of Epidemiology, University of Texas M.D. Anderson Cancer Center, Houston, Texas, USA

## Abstract

Most linkage programs assume linkage equilibrium among multiple linked markers. This assumption may lead to bias for tightly linked markers where strong linkage disequilibrium (LD) exists. We used simulated data from Genetic Analysis Workshop 14 to examine the possible effect of LD on multipoint linkage analysis. Single-nucleotide polymorphism packets from a non-disease-related region that was generated with LD were used for both model-free and parametric linkage analyses. Results showed that high LD among markers can induce false-positive evidence of linkage for affected sib-pair analysis when parental data are missing. Bias can be eliminated with parental data and can be reduced when additional markers not in LD are included in the analyses.

## Background

Most multipoint linkage programs assume linkage equilibrium among the markers being studied. This assumption is appropriate for the study of sparsely spaced markers with inter-marker distances exceeding a few centimorgans, because linkage equilibrium is expected over these intervals for almost all populations. However, with recent advances in high-throughput genotyping technology, much denser markers are available and linkage disequilibrium (LD) may exist among the markers. Applying linkage analyses that assume linkage equilibrium to dense markers may lead to bias. It is well known that misspecification of allele frequencies can cause inflation of LOD scores for both model-free [1] and model based [2,3] linkage approaches. However, estimating allele frequencies from the available data will generally correct this problem [4]. Rare exceptions such as unrecognized inbreeding at a high level or the presence of pronounced stratification might cause an excess of false-positive rates for linkage tests when only affected sib-pairs lacking parents are analyzed [5]. In the case of tightly linked loci, assuming linkage equilibrium for tightly linked markers causes incorrect inference of haplotype frequencies, which can lead to a bias similar to that induced by misspecification of allele frequencies for multi-allelic markers. However, accurately estimating haplotype frequencies is more difficult than estimating allele frequencies because of phase uncertainty. Many currently available programs such as ALLEGRO and GENEHUNTER do not allow the user to specify haplotype frequencies, while programs that will allow the user to specify haplotypes, including LINKAGE and LIPPED are very unwieldy to use in this case.

Recently, Huang et al. [6] demonstrated that assuming linkage equilibrium between tight linked markers where strong LD exists may cause apparent over-sharing of multipoint IBD among affected sibs and thus result in false-positive evidence for linkage. Here in this workshop, Genetic Analysis Workshop 14 (GAW14), we used the simulated data to further explore the effect that LD exerts in causing an excess of false-positive results. The workshop data afforded a more realistic situation upon which to study effects of LD than was covered by Huang et al. [6], because the data were simulated to represent a complex disease model and a large set of markers were available for further examination of the possible effects that LD can have upon multipoint linkage analysis.

## Methods

In order to examine the possible effect of LD on linkage analysis, we decided to study the markers from a dense marker dataset, because the inter-marker distances are smaller and the simulated LD was higher. Single-nucleotide polymorphism (SNP) packets from the non-disease related regions that were generated with LD were bought and used for the analyses. The inter-marker distance was 0.29 cM on average among these markers (20 SNPs per packet). Pedigree samples from the Aipotu population of simulated GAW14 data were used for the analyses. There were 100 nuclear families in the replicate sample and at least two sibs were affected with Kofendrerd Personality Disorder (KPD) in each family. We treated parents from each family as unrelated individuals and used them to estimate haplotype frequencies and LD. Haplotype frequencies were estimated by using the expectation maximization algorithm [7] and pair-wise LD was calculated by using standard formula [8] that are implemented in the EMLD program. We randomly selected a single sib pair from each family to ensure independence of the sib pairs. We then studied each family either including or excluding all parental genotype data. Multipoint and single-point linkage analyses of the affected sib-pair data were carried out using ALLEGRO [9]. For model-free multipoint linkage analyses, we used a Kong and Cox exponential model [10] and the score function of S_pairs _[11]. For the parametric linkage analyses, we assumed a simple dominant disease model with 100% penetrance in carriers and 0% penetrance in non-carriers, and we incorporated a heterogeneity parameter [12], thus allowing some but not all families to be linked.

## Results

Although all the SNP packets that we examined were from regions that were generated with LD, LD was not strong in most of the regions and did not have an obvious effect on linkage analysis. However, strong LD existed between three markers in SNP packet 121: B03T2407, B03T2408, and C03R0221 with pair-wise D' > 0.95 and *r*^2 ^> 0.38. The pair-wise LD as measured by D' and *r*^2 ^for this packet is shown in Table 1.

**Figure 1:**
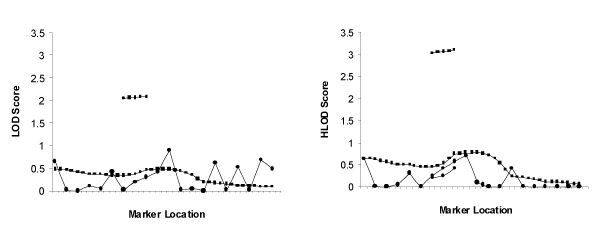
**Linkage analysis results for the 20 SNPs and the three SNPs with strong LD. **The left panel indicates results using a nonparametric NPL approach, while the right panel indicates results from a parametric linkage analysis allowing for locus heterogeneity.

Single-point linkage analysis did not show any evidence of linkage both for the three markers in strong LD alone and for the whole marker set (Fig. 1). However, using the three markers that are in strong LD and affected sib-pair only data, multipoint linkage analysis showed false-positive evidence of linkage for both model-free and parametric linkage analyses that incorporated a heterogeneity parameter (Fig. 1). This confirmed the observation by Huang et al. [6]. Including parents in the multipoint analysis eliminated the false-positive evidence (data not shown). The false-positive evidence induced by LD can be gradually reduced by adding markers that are not in LD to either or both sides of the three core markers that are in strong LD, and it seemed a better "rescue" effect can be achieved by adding markers to both sides than to a single side (Table 2). With all 20 markers, there is no evidence of linkage (maximal LOD score at the peak position: 0.34 ± 0.2).

## Conclusion

For multipoint linkage analysis of affected sib-pair data, for which parental phase information is inferred from the sib pairs, usual methods of linkage analysis assume linkage equilibrium between multiple linked markers and assigns equal probabilities to all possible phases. This assumption can cause overestimation of multipoint identity by decent (IBD) sharing and induces false positives for both model-free and parametric linkage analysis, as showed by Huang et al. [6]. This study further confirmed this observation by studying independently generated data that were simulated to reflect conditions that might be found in a genome scan. Among the markers that we studied, false-positive evidence for linkage was only obtained for a small subset of markers that showed high LD. We also showed here that including markers that are not in LD can reduce the false-positive evidence of linkage induced by markers in high LD. This indicated that including markers that are not in strong LD ensures that the haplotype frequencies are closer to those expected under the linkage equilibrium assumption and thus may help to reduce false-positive linkage findings. We also found that the LD effect is severe only when the majority of the markers being jointly examined are in strong LD. Single-point linkage analysis is not affected by LD. Therefore, given the relatively accurate allele frequencies that can readily be obtained for single marker, single-point linkage analysis can be used as a check for any suspicious false positives by comparing results to multipoint analysis. However, when a very large number of SNPs are studied, a possibility remains that allele frequency estimates for individual SNPs might be biased perhaps either by unrecognized strong stratification in the sample or by nonrandom errors introduced during processing. A potential further check is the confirmation of linkage at multiple SNPs in a region, as well as absence of linkage signal for most of the remainder of the genome. With current advances in high-throughput genotyping technology, high density marker data are easily generated. Caution must be taken when applying traditional linkage analysis to dense markers where strong LD may exist.

Our results indicate that LD among tightly linked marker should be examined, especially in the fine-mapping stage where strong LD is likely to exist between the markers. Markers that are in strong LD should not be used together for linkage analysis in order to avoid possible false positives. An alternative approach is to modify current linkage programs to allow for LD so that all marker information can be used in the search for a disease-related region.

## Abbreviations

GAW14: Genetic Analysis Workshop 14

IBD: Identity by descent

KPD: Kofendrerd Personality Disorder

LD: Linkage disequilibrium

SNP: Single-nucleotide polymorphism

## Authors' contributions

QH did the analysis and prepared the manuscript. MS assisted in the development of data for this project and performed analysis of the LD patterns of simulated data, and also presented the results at the Genetic Analysis Workshop. SS provided guidance in concept development. CIA directed the project and revised the manuscript.

## Figures and Tables

**Table 1:** Pair-wise LD between 20 SNPs of SNP packet 121 in sample replicate 1 from Aipotu population (D' measure above the diagonal and *r*^2 ^below the diagonal).

	1	2	3	4	5	6	7	8	9	10	11	12	13	14	15	16	17	18	19	20
1	--	0.653	0.278	0.130	0.211	0.234	0.085	0.214	0.358	0.154	0.004	0.138	0.093	0.254	0.053	0.392	0.546	0.068	0.057	0.163
2	0.280	--	0.309	0.062	0.141	0.206	0.227	0.369	0.493	0.048	0.109	0.021	0.053	0.023	0.049	0.115	0.151	0.127	0.228	0.116
3	0.040	0.075	--	0.272	0.103	0.018	0.218	0.064	0.160	0.349	0.198	0.083	0.134	0.393	0.177	0.047	0.332	0.046	0.450	0.227
4	0.011	0.001	0.025	--	0.223	0.016	0.122	0.772	0.690	0.475	0.397	0.103	0.084	0.375	0.272	0.162	0.371	0.480	0.385	0.051
5	0.005	0.003	0.002	0.003	--	1.000	0.408	0.524	0.325	0.001	0.095	0.000	0.012	0.121	0.062	0.225	0.058	0.026	0.006	0.010
6	0.027	0.029	0.000	0.000	0.210	--	0.081	0.427	0.365	0.224	0.196	0.264	0.212	0.113	0.194	0.121	0.093	0.048	0.408	0.098
7	0.003	0.034	0.048	0.004	0.041	0.006	--	**1.000**	**1.000**	0.216	0.052	0.139	0.241	0.005	0.019	0.169	0.140	0.068	0.085	0.117
8	0.009	0.039	0.002	0.074	0.152	0.069	**0.442**^**a**^	--	**1.000**	0.023	0.102	0.109	0.007	0.197	0.023	0.059	0.045	0.143	0.007	0.057
9	0.020	0.059	0.008	0.049	0.070	0.042	**0.396**	**0.837**	--	0.029	0.088	0.181	0.118	0.219	0.079	0.080	0.106	0.169	0.133	0.084
10	0.015	0.001	0.034	0.021	0.000	0.016	0.013	0.000	0.001	--	0.088	0.523	0.490	0.318	0.037	0.143	0.504	0.014	0.140	0.114
11	0.000	0.006	0.029	0.071	0.003	0.028	0.002	0.003	0.002	0.002	--	0.424	0.284	0.339	0.073	0.049	0.193	0.034	0.345	0.009
12	0.009	0.000	0.006	0.003	0.000	0.065	0.018	0.005	0.011	0.083	0.122	--	0.987	0.017	0.024	0.094	0.108	0.054	0.209	0.025
13	0.003	0.003	0.015	0.002	0.000	0.032	0.048	0.000	0.004	0.054	0.041	0.728	--	0.023	0.105	0.053	0.091	0.083	0.144	0.009
14	0.027	0.000	0.126	0.039	0.003	0.011	0.000	0.017	0.018	0.034	0.070	0.000	0.000	--	0.136	0.053	0.082	0.069	0.182	0.008
15	0.000	0.001	0.007	0.005	0.000	0.009	0.000	0.000	0.000	0.001	0.001	0.000	0.003	0.005	--	0.036	0.717	0.034	0.415	0.245
16	0.045	0.006	0.002	0.005	0.007	0.009	0.019	0.002	0.003	0.004	0.001	0.005	0.002	0.002	0.000	--	0.039	0.170	0.193	0.001
17	0.026	0.003	0.019	0.008	0.000	0.002	0.003	0.001	0.006	0.014	0.010	0.002	0.002	0.001	0.021	0.000	--	0.300	0.860	0.186
18	0.001	0.005	0.001	0.032	0.000	0.001	0.002	0.018	0.021	0.000	0.001	0.001	0.004	0.002	0.001	0.008	0.036	--	0.095	0.091
19	0.000	0.004	0.009	0.002	0.000	0.008	0.000	0.000	0.000	0.003	0.004	0.002	0.001	0.002	0.002	0.001	0.007	0.001	--	0.010
20	0.019	0.012	0.036	0.001	0.000	0.007	0.010	0.002	0.004	0.003	0.000	0.000	0.000	0.000	0.021	0.000	0.004	0.006	0.000	--

^a^Bold text indicates the three markers that are in strong linkage disequilibrium.

**Table 2:** Multipoint LOD scores for different set of markers from model-free linkage analysis.

Marker	Multipoint LOD scores at the marker location from model-free linkage analyses^b^							
B03T2401	--	--	--	--	--	--	--	0.48
B03T2402	--	--	--	--	--	--	--	0.46
B03T2403	--	--	--	--	--	--	--	0.42
B03T2404	--	--	--	--	--	--	--	0.36
B03T2405	--	--	1.06	--	--	--	0.69	0.35
B03T2406	--	1.06	1.08	--	--	0.78	0.73	0.33
**B03T2407**^**a**^	2.05	1.13	1.16	1.74	1.42	0.85	0.83	0.34
**B03T2408**	2.06	1.15	1.18	1.75	1.43	0.86	0.85	0.37
**C03R0221**	2.09	1.18	1.22	1.74	1.42	0.87	0.84	0.46
B03T2410	--	--	--	1.72	1.40	0.86	0.82	0.47
B03T2411	--	--	--	--	1.39	--	0.80	0.44
B03T2412	--	--	--	--	--	--	--	0.34
B03T2413	--	--	--	--	--	--	--	0.19
B03T2414	--	--	--	--	--	--	--	0.17
B03T2415	--	--	--	--	--	--	--	0.14
B03T2416	--	--	--	--	--	--	--	0.11
B03T2417	--	--	--	--	--	--	--	0.11
B03T2418	--	--	--	--	--	--	--	0.09
C03R0222	--	--	--	--	--	--	--	0.08
B03T2420	--	--	--	--	--	--	--	0.08

^a^Markers in strong LD are indicated by bold.

^b^Columns reflect results from varying multipoint analyses including markers as indicated.
